# Increased Anxiety After Stimulation of the Right Inferior Parietal Lobe and the Left Orbitofrontal Cortex

**DOI:** 10.3389/fpsyt.2020.00375

**Published:** 2020-05-05

**Authors:** Matthias Grieder, Philipp Homan, Andrea Federspiel, Claus Kiefer, Gregor Hasler

**Affiliations:** ^1^Translational Research Center, University Hospital of Psychiatry and Psychotherapy, University of Bern, Bern, Switzerland; ^2^Center for Psychiatric Neuroscience, Feinstein Institute for Medical Research, Manhasset, NY, United States; ^3^Division of Psychiatry Research, Zucker Hillside Hospital, Northwell Health, New York, NY, United States; ^4^Department of Psychiatry, Zucker School of Medicine at Northwell/Hofstra, Hempstead, NY, United States; ^5^Institute of Diagnostic and Interventional Neuroradiology, University Hospital Bern, Bern, Switzerland; ^6^Division of Psychiatry and Psychotherapy, Department of Medicine, Faculty of Science, University of Fribourg, Fribourg, Switzerland

**Keywords:** inferior parietal lobe, sustained anxiety, transcranial direct current stimulation, cerebral blood flow, arterial spin labeling

## Abstract

Sustained anxiety is a key symptom of anxiety disorders and may be associated with neural activation in the right inferior parietal lobe (rIPL), particularly under unpredictable threat. This finding suggests a moderating role of the rIPL in sustained anxiety, which we tested in the current study. We applied cathodal or sham transcranial direct current stimulation (tDCS) to the rIPL as a symptom provocation method in 22 healthy participants in a randomized, double-blind, crossover study, prior to two recordings of cerebral blood flow (CBF). In between, we applied a threat-of-shock paradigm with three conditions: unpredictable (U), predictable (P), or no electric shocks (N). We hypothesized increased anxiety under U, but not under P or N. Furthermore, we expected reduced CBF in the rIPL after tDCS compared to sham. As predicted, anxiety was higher in the U than the P and N conditions, and active tDCS augmented this effect. While tDCS did not alter CBF in the rIPL, it did attenuate the observed increase in brain regions that typically increase activation as a response to anxiety. These findings suggest that the rIPL moderates sustained anxiety as a gateway to brain regions crucial in anxiety. Alternatively, anodal tDCS over the left orbitofrontal cortex (lOFC) may have increased anxiety through disruption of OFC-amygdala interactions.

## Introduction

Imminent threat elicits so-called phasic fear, a primarily adaptive, rapidly emerging response to a specific cue that rapidly declines when the threat has disappeared. In contrast, unspecific and unpredictable threat induces anxiety (1). In other words, anxiety is triggered by potential threats that are more distant than acute threats, both physically and mentally. Altered vigilance and increased arousal have been described as consequences of anxiety (1). If such anxiety responses develop pervasive characteristics, termed as sustained anxiety, a clinical manifestation might be the consequence. Sustained anxiety is associated with psychiatric diseases such as depression, generalized anxiety disorder, and panic disorder (2), whereas exaggerated phasic fear is commonly associated with phobia and post-traumatic stress disorder (3, 4).

Theoretical and empirical debates propose that the two domains of fear and anxiety do not only share common underlying functional networks, but are characterized by a distinct interplay of fear- and anxiety-related brain regions. This view is supported by rodent data and a study with humans that revealed an involvement of frontal and inferior parietal cortical regions, which suggests that higher-order cognitive processes might play a role in sustained anxiety (5, 6). Furthermore, brain areas encompassing the amygdala and the anterior hippocampus showed altered activation responses to unpredictable threat in mood and anxiety disorders (7, 8). Of particular interest for this study is our earlier finding that the right inferior parietal lobe (rIPL) may attenuate stress in unpredictable threat situations, a notion that was based on a negative correlation between IPL activity and anxiety (9). Elevated activation in the IPL might counteract anxiety by recruiting attentional resources, which support coping with the threat situation, suggesting a role for the rIPL in anxiety resilience. This interpretation is corroborated by studies that showed an IPL involvement in visual attention, decision-making, and anxiety (10–12). The IPL is part of a dorsal fronto-parietal network for visuospatial attention, with the role of allocating attention in a top-down manner, and it has been shown that the right IPL responds to alerting stimuli in the environment (13). This view is further supported by a study by Yoshie et al. (14) who found that a social evaluation situation could alter IPL activity, which then transiently impaired sensorimotor performance. Besides, volumetric analyses involving the IPL have shown inverse relationships of IPL volume and anxiety in Alzheimer’s disease, as well as social avoidance in social anxiety disorder (15, 16). Alongside this dorsal fronto-parietal network is the ventral fronto-temporo-parietal network, which shifts attention toward salient stimuli, and acts as a so-called “circuit breaker” for the dorsal network (17). Thus, the literature suggests a theoretical framework on sustained anxiety that proposes a diversion of attention away from relevant stimuli, governed by fronto-parietal brain regions, to a sensory-vigilance mode, governed by regions such as the amygdala or the insula. Hence, rIPL activation changes would not only alter anxiety levels in subjects but would also promote activity changes in remote brain regions associated with these attention networks.

Research into functional alterations in anxiety disorders provide key information of what brain regions show such anxiety-related activation changes. For example, hyperactivation in anxiety relevant regions such as amygdala and insula is a common finding in anxiety disorders (18, 19). Moreover, the anterior cingulate cortex (ACC), ventral striatum, and ventro-medial prefrontal cortex have been found to be hypoactivated in anxiety disorders and may play an anxiety-modulating role in the anticipation of aversive stimuli (18–21). Thus, the influence of the rIPL on anxiety-related brain regions might differentially down-regulate or up-regulate anxiety-relevant networks. However, the role of the IPL, as part of the dorsal fronto-parietal network, in sustained anxiety has not been studied yet, and an involvement in resilience needs empirical evidence for further verification.

To study the role of the rIPL in anxiety, we applied transcranial direct current stimulation (tDCS), a non-invasive brain stimulation technique, as a symptom provocation method (22) to disrupt the potential resilience function of the rIPL and to increase anxiety, but not fear. To this end, we applied 20 min of cathodal stimulation in healthy individuals. Cathodal tDCS has been shown to decrease cortical excitability (23). This stimulation protocol has been shown to elicit a transient inhibition of a target region, and it has been validated and applied in various studies before [for a meta-analysis, see (24)]. Moreover, Zheng et al. (25) showed network effects of stimulation in regions functionally related to the target region. After stimulation, we assessed regional cerebral blood flow (rCBF) by arterial spin labeling (ASL) MRI, before and after an anxiety-inducing threat-of-shock paradigm. RCBF reflects an absolute measure of neuronal activation, similar to positron emission tomography (PET) methodology, in a non-invasive manner (26). We compared regional as well as network CBF between active and sham tDCS, both before and after the threat-of-shock paradigm. Using this approach rather than assessing brain activation during the threat-of-shock paradigm, we were able to disentangle the mere tDCS effect on cerebral CBF and the tDCS effect on CBF in combination with the anxiety-inducing threat-of-shock paradigm. Besides, resting rCBF is a powerful method to monitor baseline brain activation fluctuation, that is, brain activation changes that are persistent and outlast the duration of a threat situation.

We hypothesized that sustained anxiety but not phasic fear would be higher and that rIPL activation would be reduced after active compared to sham stimulation.

## Materials and Methods

### Participants

Data of 22 healthy participants (14 females) was included in this study (mean age = 26.3 years; SD = 5.4). With this sample size and the within-subject design (which increased statistical power), we were able to detect a medium to large effect (d = 0.63) with 80% power at a significance level of 5%. Note that a comparable between-subject design would have needed 41 subjects per group to achieve the same statistical power. Inclusion criteria were age (18–60 years) and normal or corrected to normal vision and hearing abilities. Exclusion criteria were left-handedness, asthma, glaucoma, current pregnancy or breast-feeding, current use of psychotropic drugs, history of drug, alcohol, and nicotine abuse within 1 year or longer than 2 years. Potential participants were also excluded if they were unable to understand the tasks and risks of the study, reported medical or neurological illness likely to affect physiology or anatomy (e.g., hypertension, cardiovascular disorders, etc.), or any lifetime major psychiatric diagnosis.

Recruitment was realized by advertisements in local newspapers, internet, and poster-bills at the University of Bern and University Hospital of Psychiatry and Psychotherapy. This study was approved by the cantonal ethics committee (“Kantonale Ethikkommission Bern”) and accorded with the Declaration of Helsinki. All participants provided informed consent for study participation and were financially compensated.

### Experimental Procedure

**Figure 1:**
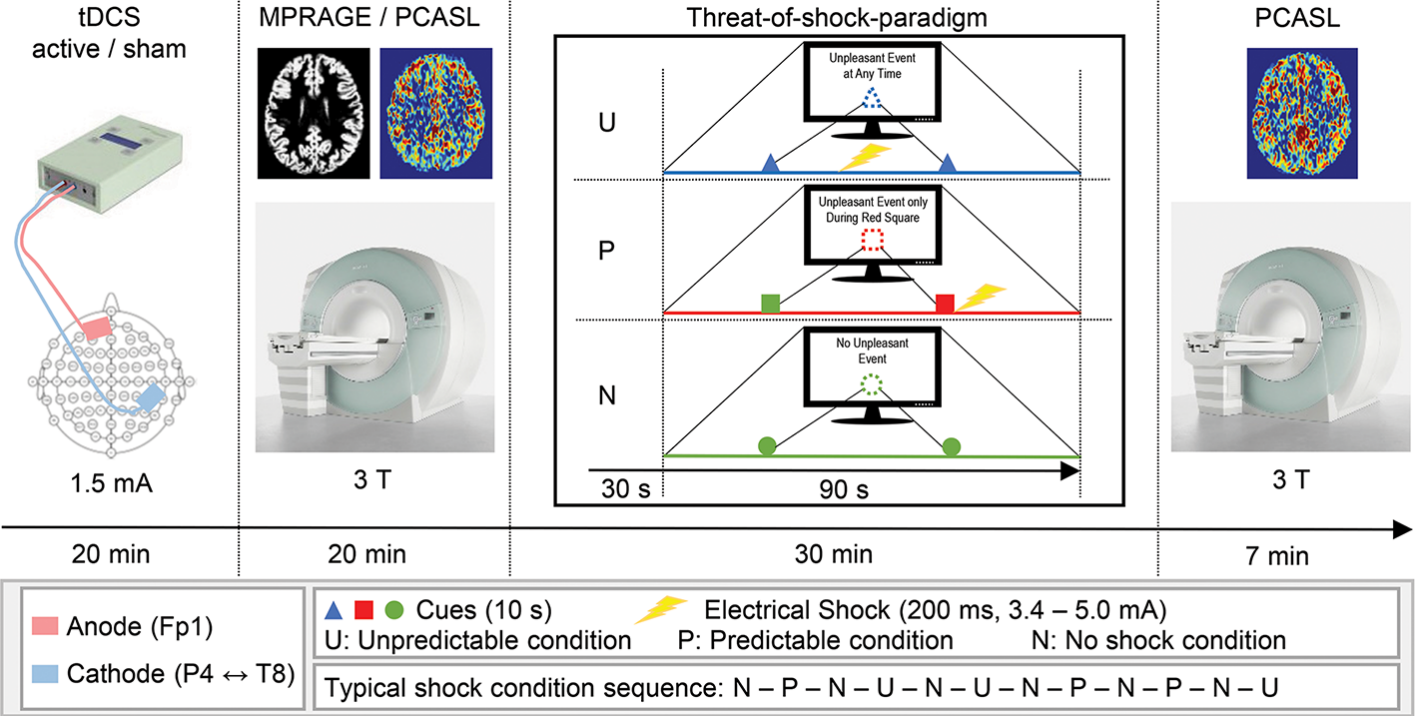
Schematic illustration of the experimental procedure. Time spent for setup and preparation is not depicted.

This randomized, double-blind, sham-controlled crossover study was conducted at Bern University Hospital (“Inselspital”) in Bern, Switzerland. Two recording sessions were scheduled for each participant at least four weeks apart from each other, with the tDCS conditions (active and sham) as the only altering factor. Each recording session was initiated by tDCS setup and 20min stimulation, where the participant sat in a comfortable chair in a quiet examination room in the hospital. After completion of the tDCS, we guided the participants to the MR facility of the hospital, where the threat-of-shock paradigm was prepared and the series of MR sequences were run (approx. 10 min between tDCS and MR scanning). Of interest for this study are the two ASL recordings, one of which was administered before the threat-of-shock paradigm, and the other afterwards (**Figure 1**). During the paradigm, a BOLD fMRI sequence was run, though due to a technical error, these data were not available.

### tDCS Protocol

A CE-certified Eldith DC-Stimulator (NeuroConn GmbH, Ilmenau, Germany) was used to apply 1.5 mA direct currents through saline water soaked sponge-coated rubber electrodes with a size of 35 cm^2^ (current density 0.43 A/m^2^). Although there is recent evidence for higher currents account better for the current density attenuating factor of soft tissue and skull, in this study, we tested a new brain region associated with anxiety and used a stimulation protocol that has been studied several times before this study (27). Moreover, we entered our planned tDCS montage into the Soterix HD-Explore software (Soterix Medical Inc., New York, NY, USA) to simulate the cortical current density distribution. The simulation yielded highest current densities over the rIPL, the right premotor cortex (rPMC), and the left orbitofrontal cortex (lOFC). Guided by the EEG 10/20-system, the center of the rIPL is located between electrodes P4 and T8, the lOFC at Fp1 (**Supplementary Figure 1**). To maintain a consistent localization of the target areas across participants, we used a standard 10/20-system EEG cap. Accordingly, the cathode was fixed with an elastic strap halfway between P4 and T8, and the anode over Fp1. In the active tDCS condition, 20 min offline DC was delivered with fade-in and fade-out ramps of 2 s each. In the sham tDCS condition, identical fade-in and fade-out ramps were applied, with an intermittent current flow duration of 15 s. This procedure of blinding the participant has been reported to be reliable (28, 29). The tDCS protocol we applied in this study was in accordance with the latest technical and safety guidelines (30).

### Threat-of-Shock Paradigm

Previous studies have demonstrated that the anticipation of an aversive event such as a non-painful electrical shock elicits a state of anxiety (9, 31). They further showed that a predictable shock (P) induces short lasting phasic fear, whereas the threat of an unpredictable shock (U) evokes anxiety, as compared to a neutral non-shock condition. These predictability-dependent specific responses have led to the development of the neutral, predictable, and unpredictable (NPU) threat experiment assessing fear and anxiety in humans using the threat of predictable and unpredictable aversive events compared to a safe condition [N, (1)], which was the basis of the paradigm used in the current study. The paradigm consisted of six electric shocks with an intensity ranging from 3.4 to 5.0 mA and a duration of 200 ms. Two disc-shaped electrodes were mounted onto the right forearm for shock administration. Individual shock intensity was determined by delivering one to three sample shocks starting at low intensity, and increasing levels until an unpleasant but not painful level was reached. During the run of the paradigm, each trial was preceded by an instruction on computer monitor that indicated one out of three threat conditions (N, P, or U). The N condition was displayed as “No Unpleasant Event”, and no shocks were delivered. In the P condition, the participants were instructed with “Unpleasant Event Only During Red Square”, and the shocks were only applied when a threat cue was depicted (i.e., variously colored geometric shapes). In the U condition, the monitor read “Unpleasant Event at Any Time”, and shocks were administered at any time. During each trial, two cues were shown for 10 s each, and a trial lasted for 1 min 30 s. The cue signaled the possibility of receiving a shock only in the P condition. In the N and U conditions, the cues had no signaling function. A trial in the conditions P or U involved one shock, which was applied with the offset of the cue in the P condition and in the absence of a cue in the U condition. One run of the paradigm comprised six trials with several minutes of rest between the runs, and two runs were assessed per participant and session. The trials were counterbalanced in that within each participant, the P and U conditions were equally likely to occur before or after an N condition. After the experiment, participants were asked to verbally rate their level of anxiety in each condition by the use of a visual analogue scale (VAS, range: 1–8).

### MRI Data Acquisition

A Siemens Magnetom Trio 3T MRI system (Siemens Medical Systems; Erlangen, Germany) was used to acquire pseudo-continuous ASL [PCASL, (32, 33)] images for CBF recording and T1-weighted anatomical images. The PCASL was a gradient-echo echo-planar sequence, acquiring images with and without labeling in an interleaved fashion. The 14 slices (voxel size = 3.4 × 3.4 × 6 mm^3^; gap = 1.5 mm) were collected in ascending order from inferior to superior. A post-label delay of 1,250 ms between the end of the labeling pulse (label time = 1,600 ms) and image acquisition (slice-acquisition time = 45 ms; FOV = 220 mm^2^; matrix = 64 × 64; TR/TE = 4,000/18 ms) was introduced. A total of 40 pairs label and control images were acquired, resulting in a total acquisition time of 5 min 20 s. Two identical PCASL acquisition runs were conducted, one before and one after the threat-of-shock paradigm. Anatomical images were acquired applying a magnetization prepared rapid gradient echo (MPRAGE) sequence (inversion time = 1000 ms; voxel size = 1 × 1 × 1 mm^3^; TR/TE = 2000/3.4 ms).

### Data Analysis

The starting point of our analysis strategy was to assess whether the threat-of-shock paradigm elicited the intended anxiety responses in the participants and thus replicated previous studies using the current paradigm. To this end, a 2 × 3 × 2 repeated measures ANOVA with stimulation (active/sham), threat condition (N/P/U), and cue (with/without) as within-subject factors was conducted.

PCASL image preprocessing was performed using routines provided by SPM12 and involved motion artefact correction by realignment, coregistration to individual anatomical T1-weighted images, and normalization into MNI space. Self-written MATLAB scripts (Version 8.5; The MathWorks Inc., Natick, MA, USA) served to quantify CBF according to the latest recommendations (34). In order to compare regional CBF changes induced by tDCS and the threat-of-shock paradigm, we extracted mean CBF values from several regions of interest (ROIs). The first ROI we defined was the tDCS target region, the rIPL. Next, we chose the contralateral homologue, the left inferior parietal lobe (lIPL) as a reference region, to assure that possible CBF effects in the rIPL do not reflect merely unspecific or global effects. Furthermore, as described in the introductory section, we aimed at disentangling possible CBF mechanisms that might mirror divergent network effects. The rationale of this approach is that one might expect that particular regions respond to the threat by increasing CBF, while others lowering CBF for compensating or regulating purposes. We composed the anxiety networks with ROIs based on a meta-analysis of Etkin and Wager (19), and findings of a study from Jensen et al. (20). Accordingly, our primary anxiety network consisted of bilateral amygdalae and insular cortices. These regions commonly show increased activation as a response to fear and anxiety. The secondary anxiety network included bilateral caudate nuclei and putamen, anterior cingulate cortices, gyri recti, and orbito-medial frontal cortices. Bishop (35) reported that with higher anxiety, especially the involved frontal regions appear to show a reduced neuronal response. Lastly, the anode region (lOFC) and the rPMC served as ROIs as well, since the current flow simulation showed elevated current densities (**Supplementary Figure 1**), which might alter CBF. The ROIs and the anxiety networks were retrieved using automated anatomical labeling (aal) in the WFU Pickatlas tool [Version 3.0.5, (36–38)]. A 2 × 2 repeated measures ANOVA with stimulation (active/sham) and run (#1 = after active or sham and before task/#2 = after active or sham and after task) as within-subject factors was conducted to investigate CBF changes of tDCS and time point. Both repeated measures ANOVA as well as *post hoc* tests were performed with the SPSS software (version 24, IBM Corp., Armonk, NY, USA). Three-dimensional ROI visualization was accomplished using MRIcroGL (http://www.mccauslandcenter.sc.edu/mricrogl).

## Results

### Self-Reported Anxiety

**Figure 2:**
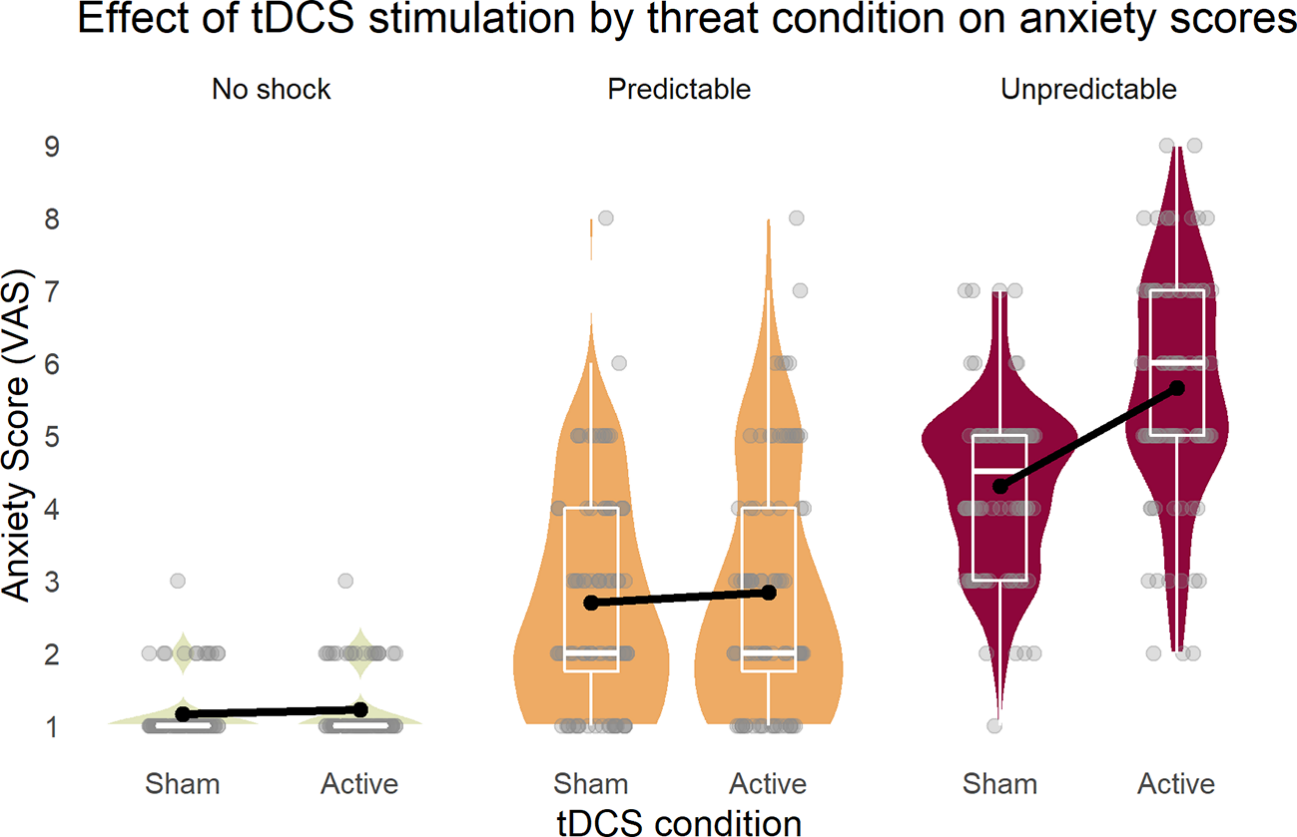
Inferior parietal stimulation by tDCS specifically increased anxiety as response to unpredictable shocks. Boxplots on the background of violin graphs illustrate two-way interaction of stimulation × condition as well as the threat condition main effect. Anxiety scores were highest in U, and P was higher than N, regardless of the tDCS condition. Active tDCS increased anxiety only in the U threat condition. U, unpredictable; P, predictable; N, neutral.

The repeated measures ANOVA (sphericity assumed: Mauchly-W(2) = 0.88, p = 0.27) yielded a three-way interaction of stimulation, threat condition, and cue [F(2,42) = 4.24, p = 0.02, partial η^2^ = 0.17]. To disentangle this interaction, all main effects and two-way interactions are reported. First, all three main effects were significant [stimulation: F(1,21) = 28.3, p < 0.001, partial η^2^ = 0.57; condition (Huynh-Feldt corrected): F(1.6,34.0) = 207.15, p < 0.001, partial η^2^ = 0.91; cue: F(1,21) = 71.3, p < 0.001, partial η^2^ = 0.77]. Next, while the two-way interactions of stimulation × condition [**Figure 2**, Greenhouse-Geisser corrected: F(1.5,32.2) = 27.6, p < 0.001, partial η^2^ = 0.57] and condition × cue [Greenhouse-Geisser corrected: F(1.3,43.5) = 61.7, p < 0.001, partial η^2^ = 0.75] were significant, stimulation × cue showed no effect [F(1,21) = 1.0, p = 0.33, partial η^2^ = 0.05)] These results illustrated that the tDCS stimulation increased self-reported anxiety in the U (unpredicted) threat condition, but not in the others.

**Figure 3:**
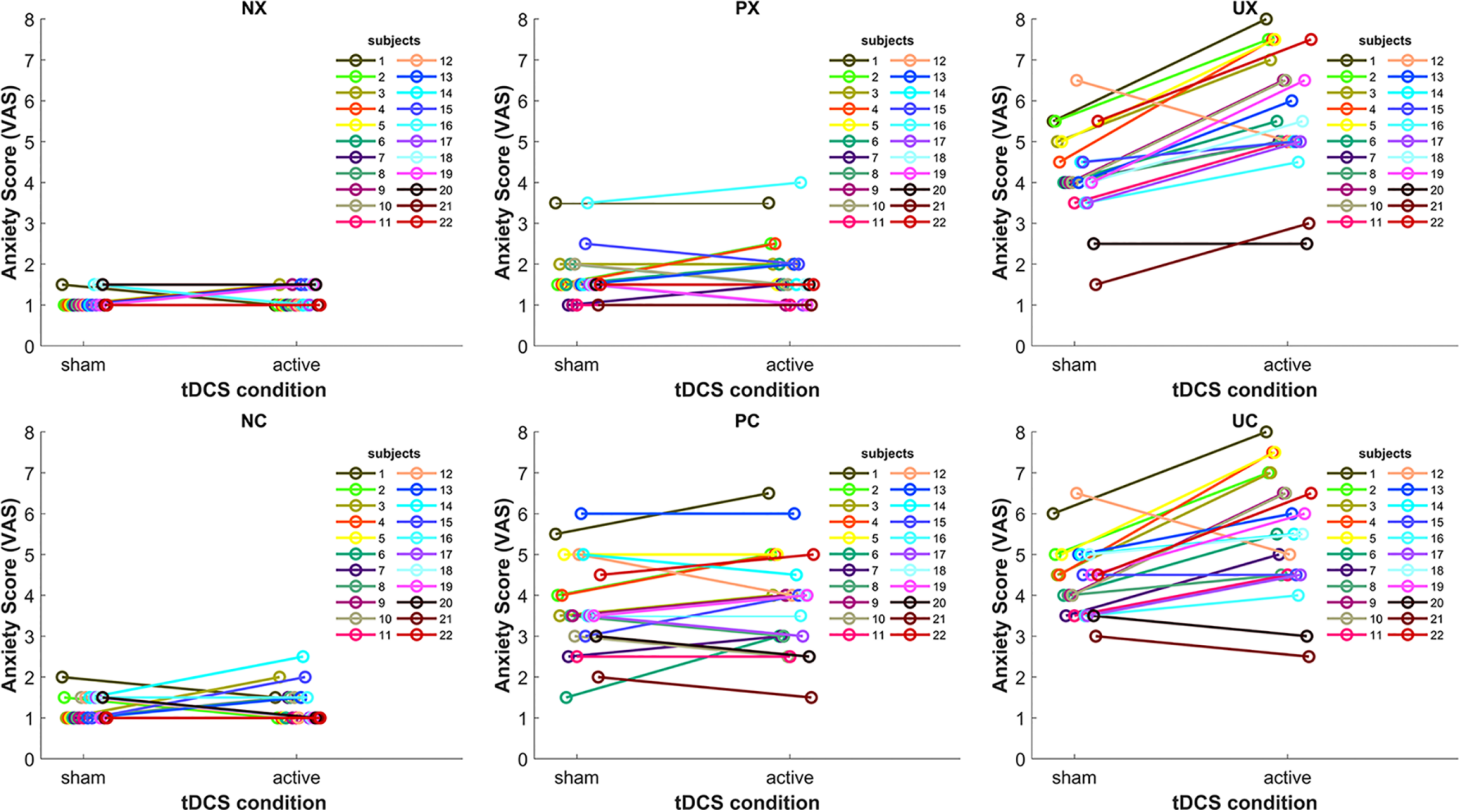
Line plots depicting the tDCS effect on anxiety in the six threat conditions for each subject. NX, neutral no cue; NC, neutral with cue; PX, predictable no cue; PC, predictable with cue; UX, unpredictable no cue; UC, unpredictable with cue.

Because previous tDCS studies showed high inter-subject variability of cortical responses to tDCS (39–41), we inspected individual stimulation anxiety provocation effects in the different threat-of-shock conditions (**Figure 3**). Individual results confirmed the group statistics. In the neutral (N) threat condition, we might see two tDCS responders (subjects 14 and 15), who reported a slightly increased anxiety in the NC condition in active versus sham. In the predictable (P) threat condition, there were five subjects with an anxiety increase of more than 0.5 on the VAS in active versus sham. No other subjects exhibited any kind of tDCS-related change. In contrast, in the unpredictable (U) threat condition, all subjects but four (subjects 12, 20, and 17, 21 in UC only) responded to the active tDCS stimulation with increased anxiety.

### Cerebral Blood Flow

**Figure 4:**
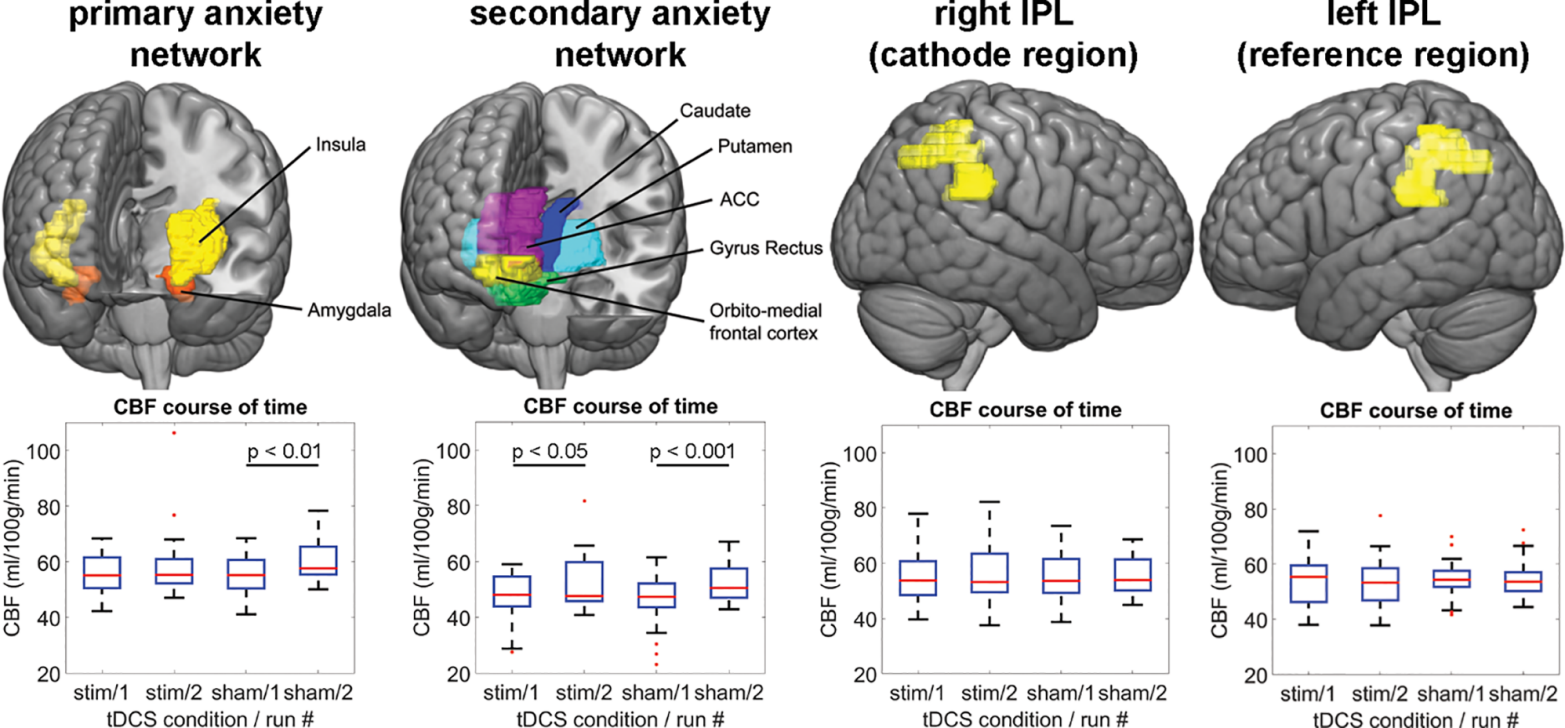
The upper panel illustrates the ROIs, for which the CBF-analysis was performed. The lower panel shows the regional CBF distribution for each ASL run per tDCS condition and network/region. IPL, inferior parietal lobe; CBF, cerebral blood flow; ACC, anterior cingulate cortex; stim, active tDCS.

**Figure 4** shows the mean CBF across subjects of each PCASL run for the anxiety networks as well as the tDCS target region rIPL and the reference region lIPL (#1 = after active/sham and before task. #2 = after active/sham and after task). For the primary anxiety network, the repeated measures ANOVA yielded a two-way interaction of stimulation and run [F(1,19) = 4.59, p = 0.045, partial η^2^ = 0.19]. There was a main effect of run [F(1,19) = 5.89, p = 0.025, partial η^2^ = 0.24], but not of stimulation. In order to disentangle these results, we computed non-parametric *post hoc* Wilcoxon tests for run in both stimulation conditions. There was a significant difference of the CBF between run one in the sham condition (Z = −2.69, p = 0.007), but not in the stimulation condition (Z = −1.09, p = 0.277). For the secondary anxiety network, the repeated measures ANOVA yielded a two-way interaction of stimulation and run [F(1,19) = 7.84, p = 0.011, partial η^2^ = 0.29]. There was a main effect of run [F(1,19) = 11.87, p = 0.003, partial η^2^ = 0.38], but not of stimulation. The *post hoc* Wilcoxon tests for this sub-network showed an increase of CBF in the second runs in both stimulation conditions as compared to the first runs (active: Z = −2.48, p = 0.013; sham: Z = −3.55, p < 0.001). Additional repeated measures ANOVA yielded no two-way interactions of stimulation and run in the lOFC [F(1,17) = 0.41, p = 0.95, partial η^2^ = 0.001] or the rPMC [F(1,19) = 0.25, p = 0.62, partial η^2^ = 0.013]. However, a main effect of run in the lOFC [F(1,17) = 6.91, p = 0.018, partial η^2^ = 0.29] and a trend in the rPMC were found [F(1,19) = 3.91, p = 0.06, partial η^2^ = 0.17; **Supplementary Figure 2**]. Taken together, this analysis indicated that the threat-of-shock paradigm induced a CBF increase in both anxiety sub-networks and the lOFC. However, the stimulation inhibited such an increase in the primary anxiety network, but not in the secondary anxiety network or the lOFC. For the rIPL, lIPL, and rPMC, the repeated measures ANOVA did not yield any effect. The outcome of participant blinding is reported in **Supplementary Table 1**.

## Discussion

Our study is consistent with previous reports on a moderating role of the rIPL in human anxiety response to unpredictable threat. We observed increased sustained anxiety but not phasic fear ratings after active compared to sham tDCS over the rIPL in our healthy participant sample. Thus, our behavioral results suggest a role for the rIPL in resilience to sustained anxiety, where elevated activation in the rIPL mitigates experienced anxiety and vice versa. Moreover, the neurobiological results may indicate that the rIPL acts as a functional gateway to brain regions that increase activation during anxiety. Alternatively, we could interpret our simulation data as evidence that anodal tDCS over the left orbital region contributed to the observed outcome changes because it changed the absolute error estimation value of the orbital region.

### Self-Reported Anxiety

The starting point of our study was the anxiety score of the three experimental conditions in the threat-of-shock paradigm. Based on Hasler et al. (9), we predicted higher anxiety in the U than the P and N condition. Indeed, we found highest anxiety in the U, and higher anxiety in the P than the N condition, a pattern that has been described also by Grillon et al. (31). Moreover, the cue reinforced anxiety in P, but not in the other conditions (5, 9). Yet most importantly, cathodal tDCS over the rIPL combined with anodal lOFC stimulation led to higher anxiety ratings in the unpredictable threat condition only by suppressing the resilient contribution of the rIPL in the processing of the threat or by stimulating the lOFC, which plays an important role in sustained anxiety through interactions with the amygdala (42). Besides, the stimulation had no effect on the factor cue as expected, since the cue was relevant only in the fear (i.e., P) condition, and the active tDCS was supposed to selectively target anxiety (i.e., U). A difference to previous studies was found in the P condition with cue, which scored lower than any U condition in our study, instead of highest as in the other studies. A possible reason for this discrepancy might be that in our study, participants provided the anxiety ratings after the experiment, when the acute phasic threat of the P condition was over and possibly not perceived as threatening as during the experiment.

### Cerebral Blood Flow

We measured CBF with ASL-MRI to identify the anxiogenic effect of inhibitory rIPL stimulation combined with anodal lOFC stimulation on a metabolic level. Our main hypothesis was a decreased CBF in the rIPL after active tDCS compared to sham. In contrast, our CBF data did not confirm such a simple, causal model of the rIPL in sustained anxiety. Instead, the CBF data suggested that the rIPL-induced resilience against the anxiety caused by the unpredictable threat is engaged only during the awareness of a threat, but not in its absence. Note that during the CBF-recording, there was no threat condition present. Moreover, active tDCS over the rIPL was related to a CBF reduction in our primary anxiety network including amygdala and insula, and thus acted as a modulator to these regions, rather than holding an anxiety-specific function. In addition, anodal lOFC stimulation may have contributed to our findings, given the important OFC-amygdala interaction in sustained anxiety. In a study that investigated the certainty-effect of a threat, the insula was more strongly activated in a condition of a certain as compared to uncertain pain threat. On the other hand, the rIPL was more strongly activated in the uncertain as opposed to the certain pain condition (43). Thus, the authors of this study as well as findings in other studies support the view that the rIPL modulates attentional top-down processes and thus regulates activation in subcortical brain regions (11, 44). Such a network effect without actual activation change in the stimulated area would require these regions be functionally connected. In a recent repetitive transcranial magnetic stimulation study, Tik et al. (45) found that stimulation of the dorsolateral prefrontal cortex increased functional connectivity in one out of nineteen resting-state networks. This particular network encompassed the DLPFC, the ACC, and the inferior parietal lobe amongst others. Hence, beside the indication of an existent functional connectivity between the stimulation area of our study (rIPL) and anxiety relevant regions, Tik’s study stressed the importance of the brain’s connectome when interpreting brain stimulation effects.

Active tDCS also modulated the secondary anxiety network, however to a lower degree by attenuating the CBF increase, as the two-way interaction of stimulation and run indicated. The effect might have mirrored relief by the participants after having been released from the threat-of-shock paradigm, because the caudate nucleus and the putamen are part of the reward system (20). Hence, the weaker tDCS effect compared to the primary anxiety network effect (which did not involve relief-related brain regions) could have been owed to relief that was stronger than the potentially outlasting anxiety activity.

### Synthesis

Interpreting the behavioral and metabolic effects of the present study, the CBF effect appeared to be linked with the tDCS-reinforced anxiety scores, as the lower CBF in amygdala and insula was the consequence of active tDCS and the threat-of-shock paradigm, revealed by the two-way-interaction and *post hoc* tests of the ANOVA on the CBF data. This CBF decrease was detected after completion of the threat-of-shock paradigm and therefore mirrored the aspect of pervasiveness in sustained anxiety.

Although we did not postulate any *a priori* assumptions of CBF effects of task and tDCS in the subcortical brain regions, one might have expected a CBF increase in the primary anxiety network after tDCS rather than a decrease. However, bearing in mind that our participants were healthy, we have to assume that their neurophysiological threat response was functional. Therefore, the CBF increase at least in the primary anxiety network in the sham tDCS condition reflected a healthy experience of anxiety or coping with anxiety (induced by the threat-of-shock paradigm). With the active tDCS interference on brain regions relevant to anxiety, the healthy resilience response was deteriorated and reported anxiety increased. As described above, hyperactivity in anxiogenic brain regions is found in patients showing sustained anxiety symptoms (18, 19). We therefore showed that in healthy participants, an increased CBF in amygdala and insula is not a necessity to reinforce anxiety, but rather the interference *per se*.

### Limitations

A few critical issues that the current study might have raised are briefly discussed. First, the common tDCS montage that we adopted for this study involved not only the cathode over the rIPL, but also the anode (commonly referred as the reference electrode) over the left orbital region. Considering the proximity of this location to brain regions involved in anxiety processing and a possible anodal stimulation effect, one cannot rule out any excitatory effects due to the active stimulation (46). For example, Heeren et al. (47) showed decreased attentional bias after anodal tDCS over the dorsolateral prefrontal cortex. Thus, the anxiogenic effect we discovered might have been modulated by the anodal tDCS over the orbital region or ultimately caused by the anode only. In future studies, the use of extracephalic reference electrodes and/or placement of electrodes over other cortical areas that are less related with anxiety (e.g., the left primary motor cortex) may increase anatomical precision of our findings. Moreover, to maximize focal precision of the stimulation, future studies could take advantage of a neuronavigation technique. At this point, it is worth emphasizing that the exact mechanisms of tDCS are still only poorly understood. However, the effect sizes appear to be sufficiently large to consider tDCS as a clinically relevant intervention tool, as for instance shown in a meta-analysis by Brunoni et al. (48). Second, one might argue that the amygdala is involved in phasic fear as well as anxiety processing (6), and that the CBF change we found might not be specific to anxiety only. However, since the active tDCS only affected anxiety in the U condition (and not the fear condition P), we are confident that our CBF effect was related to anxiety rather than fear. With respect to the CBF effects (or the lack of a CBF effect in the rIPL), a higher stimulation current might have had a higher impact on CBF levels (27). Finally, an active control condition (stimulation of a non-relevant brain region) could have corroborated the relationship of the rIPL stimulation and increased anxiety. Nevertheless, the specific behavioral effect (increased anxiety in U but not P or N after active tDCS) indicated a relationship between rIPL stimulation and anxiety.

## Conclusion

This study suggests the rIPL as an anxiety-modulating brain region in the face of unpredictable threat. By transiently inhibiting the rIPL, anxiety but not fear was reinforced. Alternatively, the anodal stimulation of the lOFC may have enhanced sustained anxiety, which is consistent with an important role of OFC-amygdala interactions in trait anxiety. Since anxiety rather than fear underlies major psychiatric conditions such as panic disorder, generalized anxiety disorder, posttraumatic stress disorder and depression, our results may have important and far-reaching clinical implications. In particular, our findings might encourage future research to examine tDCS’s potential therapeutic effects in mood and anxiety disorders.

## Data Availability Statement

Data as well as the data processing subroutines can be requested from the corresponding author.

## Ethics Statement

The study involved human participants and was reviewed and approved by Kantonale Ethikkommission Bern. The participants provided their written informed consent to participate in this study.

## Author Contributions

MG: collected the data, conceptualized and performed data and statistical analyses, and wrote the manuscript. PH: designed the study, collected the data, conceptualized and performed data and statistical analyses, and wrote the manuscript. AF: conceptualized and performed data and statistical analyses. CK: collected the data. GH: designed the study and wrote the manuscript. All authors contributed to discussion about interpretation of the results, revised, and approved the final version of the submitted manuscript.

## Funding

This work was supported by the University of Bern. The funding source was not involved in study design, data collection, analysis, or interpretation of data.

## Conflict of Interest

The authors declare that the research was conducted in the absence of any commercial or financial relationships that could be construed as a potential conflict of interest.
