# Supplementary figures and images for: Increased Anxiety After Stimulation of the Right Inferior Parietal Lobe and the Left Orbitofrontal Cortex
Source: Front Psychiatry. 2020 May 5;11:375. doi: 10.3389/fpsyt.2020.00375 (PMC7214722; doi:10.3389/fpsyt.2020.00375)

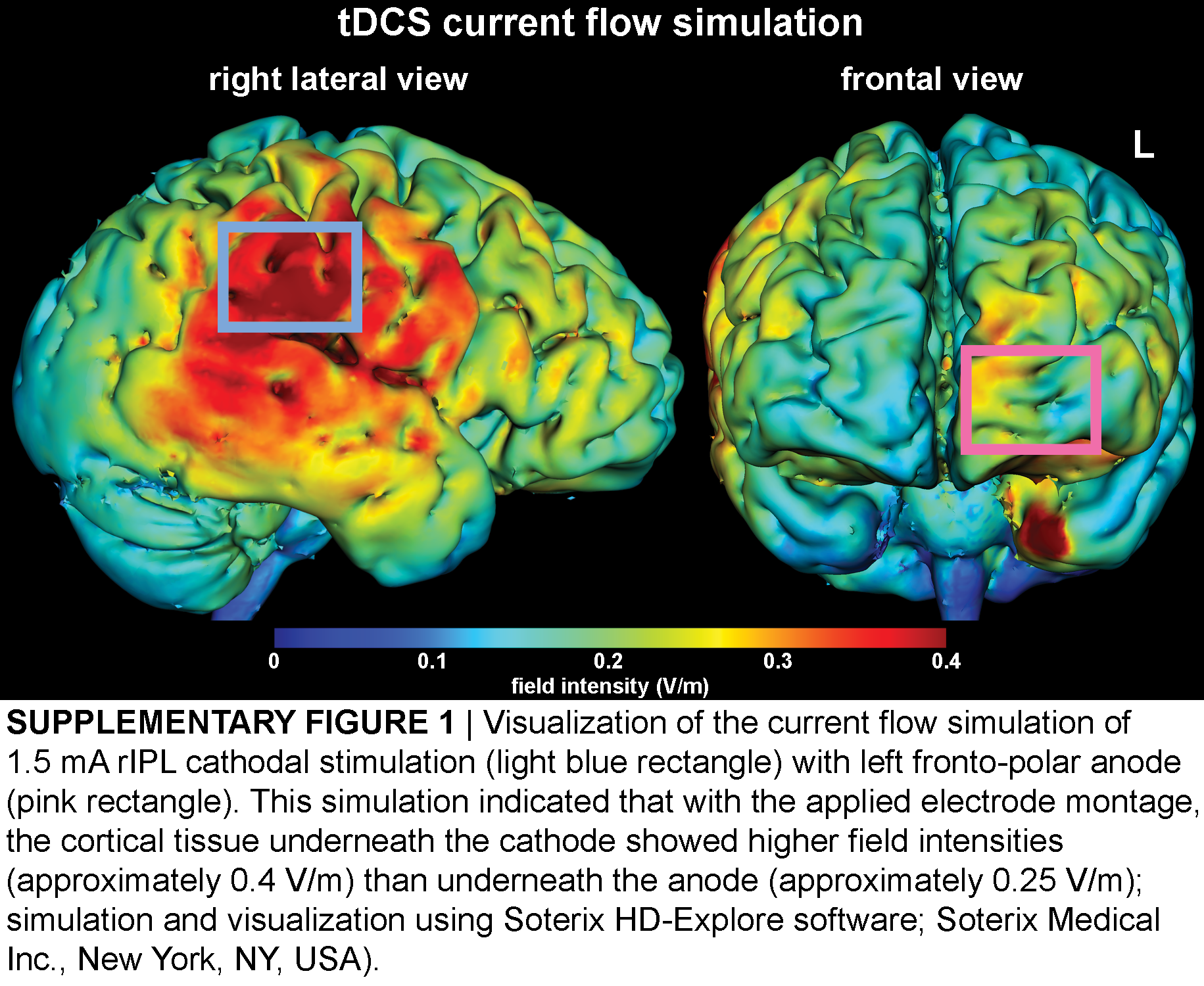

Supplement: Supplementary file 1 [file Image_1.tif]

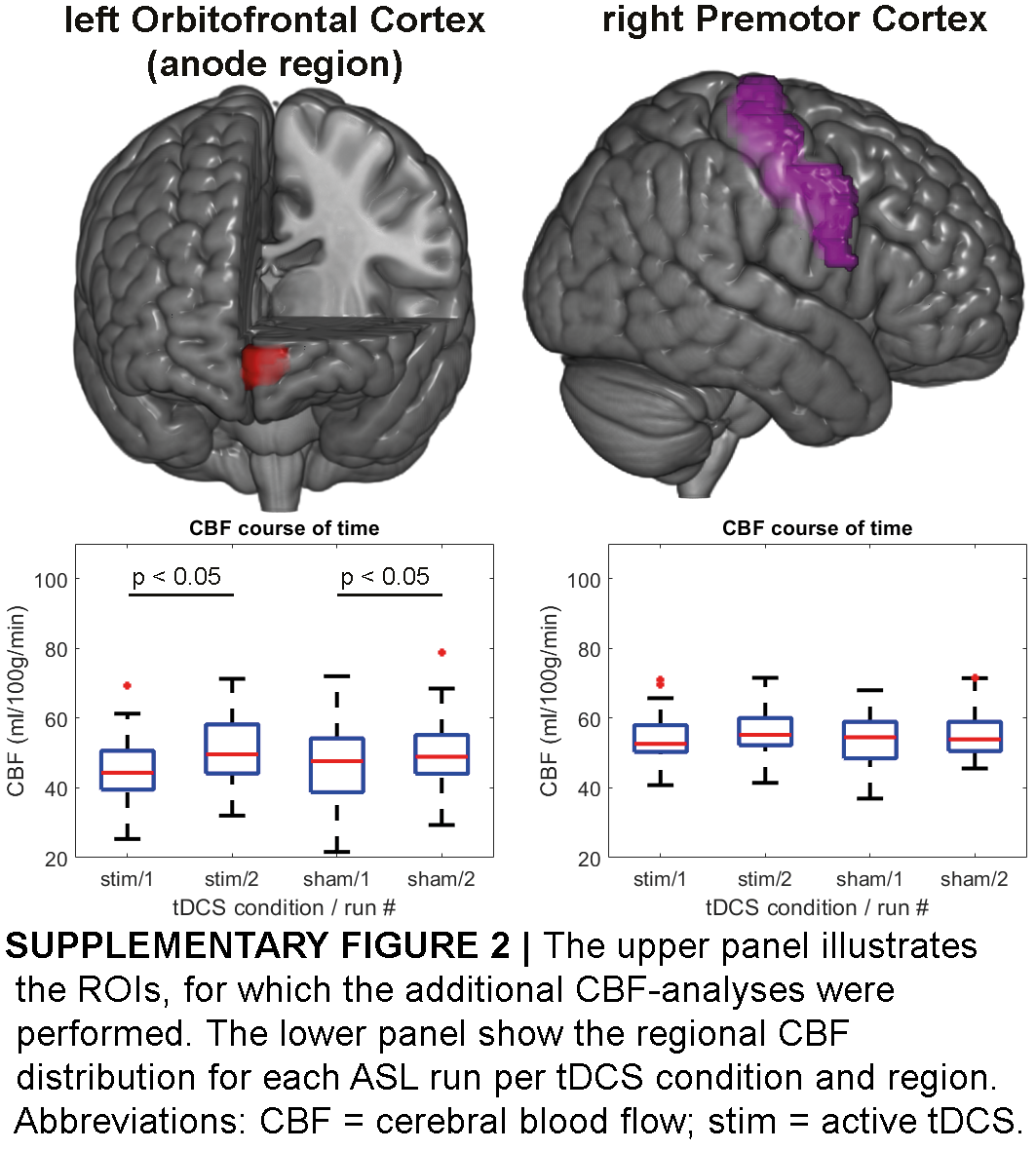

Supplement: Supplementary file 2 [file Image_2.tif]
