# Supplementary material for: Increased Anxiety After Stimulation of the Right Inferior Parietal Lobe and the Left Orbitofrontal Cortex
Source: Front Psychiatry. 2020 May 5;11:375. doi: 10.3389/fpsyt.2020.00375 (PMC7214722; doi:10.3389/fpsyt.2020.00375)
Supplement: Supplementary file 3 [file Table_1.docx]

Supplementary Material

# Supplementary Table 1

Numbers and percentages of the participants who correctly identified the stimulation condition (Identified) or both conditions correctly (All correct). Compared with Ambrus et al. (2012), our participants were less likely to correctly identify the active stimulation condition. Note: stimulation condition guess of one participant was missing in the sham condition.

|  |  | Identified | | All correct | |
| --- | --- | --- | --- | --- | --- |
|  |  | N | % | N | % |
| Naïve N = 22 | Active | 9 | 40.9 | 3 | 14.2 |
| N = 21 | Sham | 6 | 28.6 |  |  |
